# The Effect of Cholecystectomy on the Risk of Colorectal Cancer in Patients with Gallbladder Stones

**DOI:** 10.3390/cancers12030550

**Published:** 2020-02-27

**Authors:** Chien-Hua Chen, Cheng-Li Lin, Chia-Hung Kao

**Affiliations:** 1 Digestive Disease Center, Changbing Show-Chwan Memorial Hospital, Lukang Township, Changhua County 500, Taiwan; showchench@yahoo.com.tw; 2 Digestive Disease Center, Show-Chwan Memorial Hospital, Changhua 500, Taiwan; 3 Department of Food Science and Technology, Hungkuang University, Taichung 433, Taiwan; 4 Chung Chou University of Science and Technology, Yuanlin Township, Changhua County 500, Taiwan; 5 Management Office for Health Data, China Medical University Hospital, Taichung 404, Taiwan; orangechengli@gmail.com; 6 College of Medicine, China Medical University, Taichung 404, Taiwan; 7 Graduate Institute of Biomedical Sciences and School of Medicine, College of Medicine, China Medical University, Taichung 404, Taiwan; 8 Department of Nuclear Medicine and PET Center, China Medical University Hospital, Taichung 404, Taiwan; 9 Department of Bioinformatics and Medical Engineering, Asia University, Taichung 404, Taiwan; 10 Center of Augmented Intelligence in Healthcare, China Medical University Hospital, Taichung 404, Taiwan; 11 Graduate Institute of Biomedical Sciences and School of Medicine, College of Medicine, China Medical University, Taichung 40447, Taiwan

**Keywords:** cholecystectomy, colorectal cancer, gallbladder stones, cohort study

## Abstract

To evaluate the risk of colorectal cancer (CRC) after cholecystectomy for gallbladder stones (GBS). Methods: This nationwide population-based cohort study analyzed the inpatient data from the Taiwan National Health Insurance Research Database. The study cohort comprised of 83,963 patients aged ≥ 20 years undergoing cholecystectomy for GBS between 2000 and 2010. The control cohort comprised the GBS patients without cholecystectomy, who were propensity matched with the study cohort at a 1:1 ratio based on age, sex, comorbidities, and the index date for cholecystectomy. Results: The cumulative incidence of CRC within 6 months of follow-up was higher in the cholecystectomy cohort than that in the non-cholecystectomy cohort (aHR (adjusted hazard ratio) = 7.90, 95% confidence interval (CI) = 6.27–9.94; log-rank test, *p* < 0.001). The cumulative incidence of CRC after 6 months of follow-up was lower in the cholecystectomy cohort than that in the non-cholecystectomy cohort (aHR = 0.66, 95% CI = 0.60–0.73; log-rank test, *p* < 0.001), but the reduced risk of CRC for the cholecystectomy cohort was statistically significant only in rectal cancer after separately considering females (aHR = 0.64, 95% CI = 0.46–0.88) and males (aHR = 0.59, 95% CI = 0.44–0.79). Conclusions: The positive association between cholecystectomy and the CRC risk within the first 6 months after cholecystectomy might be due to a detection bias or pre-existing CRC. However, cholecystectomy is associated with a decreased risk of rectal cancer, rather than proximal or distal colon cancer, after more than 6 months of follow-up.

## 1. Introduction

Colorectal cancer (CRC) ranks as the third most common malignancy for men and the second most common malignancy for women worldwide [1]. Moreover, CRC remains as the fourth most common malignancy-associated mortality globally [1,2]. With global industrialization and urbanization, approximately 1,800,000 new cases and 881,000 deaths have been diagnosed with CRC in 2018 globally although the CRC mortality has decreased for the last decades in Western countries [1,3]. In Taiwan, the age-standardized incidence rate of CRC has increased from 25.46/100,000 persons in 2002 to 43.0/100,000 persons in 2015 [4]. Moreover, the age-standardized mortality rate of CRC was 11.6/100,000 persons in 2017 and CRC has remained as the third commonest malignancy-associated mortality in Taiwan [4]. Therefore, it is important to identify the predisposing factors and the interventional strategies for the prevention of CRC.

Cholelithiasis is a common disease for the adult population, and gallbladder stones (GBS) accounts for approximately 85% of the stone distribution for cholelithiasis [5]. The reported prevalence of GBS was approximately 10–20% in Western countries and 5–10% in Taiwan, respectively [6,7,8]. It has been supposed that GBS is associated with the development of CRC due to chronic inflammation, increased secretion of secondary bile acid in both GBS and CRC, and shared common risk factors between GBS and CRC [9,10]. The effect of cholecystectomy in contributing to the development of CRC remains controversial since it can decrease the oxidative stress from the gallbladder to diminish the risk of CRC and it may increase secretion of the bile juice into the bowels to enhance the development of CRC [11].

No association between cholecystectomy and CRC was observed in a former meta-analysis by analyzing the cohort and case-control studies [12]. By contrast, a positive aforementioned association was observed in a recent meta-analysis based on 10 cohort studies [13,14]. However, we find that all the control cohorts are in the general population, rather than the patients with GBS; therefore, the contribution of GBS to the development of CRC might have been overlooked in the former studies. The association between cholecystectomy and CRC for the GBS patients has been rarely discussed in the literature. In this study, we hypothesized that cholecystectomy is associated with a decreased risk of CRC in GBS patients based on the former studies supporting GBS is related to the development of CRC [9,10]. This nationwide population-based cohort study analyzed the hospitalization claims data, a claims data for hospitalization of the National Health Insurance (NHI) program, of the Taiwan National Health Insurance Research Database (NHIRD) to evaluate the risk of CRC after cholecystectomy in GBS patients. We also follow up the duration effect on the aforementioned association to avoid the bias caused by the temporal effect.

## 2. Methods

### 2.1. Data Source

Taiwan has launched this government-owned NHI program since 1 March, 1995 and this mandatory program has covered almost every resident of the 23.74 million population in Taiwan. We analyzed the inpatient data of the NHIRD, which contains claims data for hospitalization of the NHI program in Taiwan, and classified the disease codes by the 2001 International Classification of Diseases, Ninth revision, Clinical Modification (ICD-9-CM) in our study [15,16,17].

### 2.2. Ethics Statement

The National Health Research Institute (NHRI) of Taiwan is authorized by the Taiwan government to be in charge of the administration of NHIRD, which has encrypted personal information to protect privacy. Furthermore, the researchers can apply the NHIRD to conduct the medical studies after approval from NHRI. Therefore, getting patient consent is waived for accessing the NHIRD and this study has been approved by the Institutional Review Board (IRB) of China Medical University (CMUH104-REC2-115-CR4).

### 2.3. Data Sharing Statement

The Taiwan Ministry of Health and Welfare (MOHW) approved our application to access this database. The contact information for MOHW is the following email: stcarolwu@mohw.gov.tw, address: No.488, Sec. 6, Zhongxiao E. Rd., Nangang Dist., Taipei City 115, Taiwan (R.O.C.), and phone: +886-285-906-848.

### 2.4. Sampled Patients

**Figure 1:**
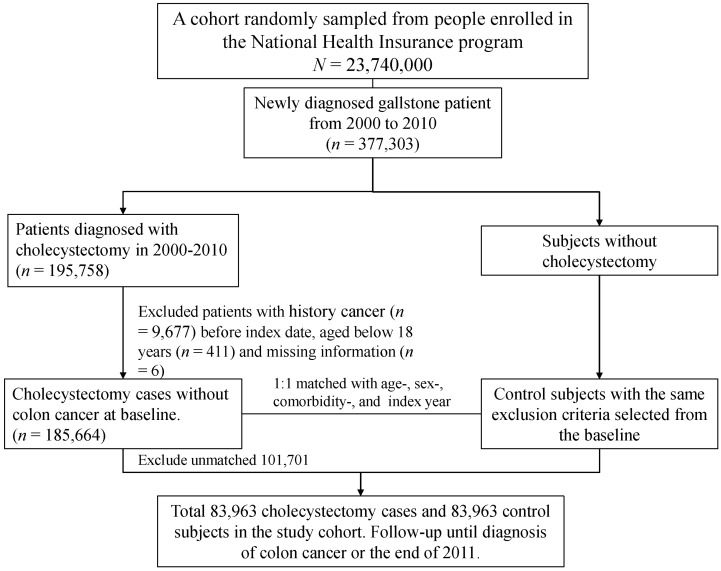
The selection process for the gallbladder stones patients with and without cholecystectomy in the 2 study cohorts.

The selection process for both cohorts with GBS (ICD-9 code 574.0, 574.1, 574.2, 574.6, 574.7, 574.8, and 574.9) is shown in Figure 1. This study enrolled GBS patients aged ≥ 20 years, who underwent cholecystectomy between 1 January 2000 and 31 December 2010. Patients with cholecystectomy were matched (1:1 ratio) with those who did not have cholecystectomy according to their propensity score through nearest neighbor matching, initially to the eighth digit and then as required to the first digit. Therefore, matches were first made within a caliper width of 0.0000001, and then the caliper width was increased for unmatched cases to 0.1. We reconsidered the matching criteria and performed a rematch (greedy algorithm). For each GBS patient underwent cholecystectomy, the corresponding comparisons were selected based on the nearest propensity score. The propensity score was calculated using the probability of the cholecystectomy assignment by using a logistic regression model and included the following baseline variables: age, gender, occupation, urbanization level, comorbidities of hypertension, hyperlipidemia, cirrhosis, stroke, CAD, diabetes mellitus, COPD, chronic kidney diseases, alcohol-related illness, colorectal adenomas, inflammatory bowel disease, irritable bowel syndrome, obesity, and index year. It has been reported that GBS is associated with colorectal adenoma, but no association with cholecystectomy [18]. It will be more appropriate to regard colorectal adenoma as the predictor of CRC, although adenoma may be the possible mediators of CRC. Moreover, the pathogenesis relating GBS with CRC remains unknown and it is not necessarily through the progression of adenoma. We, hence, still match colorectal adenomas for both cohorts to focus on the association of cholecystectomy with CRC, rather than colonic neoplasm. Rather than the date for diagnosis of GBS, the index date appointed for the control cohort to be followed up was assigned based on the date for cholecystectomy in the case cohort. The comorbidities considered for analysis in this study included hypertension, hyperlipidemia, cirrhosis, stroke, coronary artery disease (CAD), diabetes, chronic obstructive pulmonary disease (COPD), chronic kidney injury, alcohol-related illness, colorectal adenoma, inflammatory bowel disease, irritable bowel syndrome, and obesity. We did not include the patients with a history of malignancy (ICD-9-CM 140–208) or incomplete information of age or sex in both cohorts. We followed up each patient from the index date until the development of CRC (ICD-9 code 153 and 154), death, withdrawal from the NHI program due to emigration or death, or the end of 31 December 2011. The deaths would be censored if the causes of deaths are unidentified. We classified level 1 as the highest urbanized area and level 4 as the lowest urbanized area accordingly by the population density, education level of residents, elderly and agricultural population density, and the density of physicians [19]. The white-collar occupations consisted of indoor works such as government functionary, education, or administration in business or industries. The blue-collar occupations consisted of labor works in agriculture, fishery, or industries. We categorized the individuals who are primarily retired, unemployed, or subsidiary as those with other occupations [20].

### 2.5. Statistical Analysis

The chi-squared test was used to examine the categorical analysis between the cholecystectomy and non-cholecystectomy cohorts. The Student’s *t* test was used to compare the continuous variables, such as mean ages (standard deviations (SDs)) and mean follow-up period (SDs), between the cohorts. We compared the cumulative incidence of CRC events and survival, within and more than 6 months after cholecystectomy, between the cohorts by the Kaplan–Meier method and examined the differences by the log-rank test. We estimated the CRC incidence rates by stratifying age, sex, occupation, urbanization, and the presence or absence of a comorbidity. We assessed the risk (hazard ratios (HRs) and 95% confidence intervals (CIs)) of CRC by univariable and multivariable Cox proportional hazard regression models. We only included those variables significant in the univariable analysis for further examination in the multivariable analysis. The software used for analysis is SAS Version 9.4 (SAS Institute, Cary, NC, USA) and it is considered as statistical significance when a two-tailed *p* is <0.05. To compare the colon cancer incidence densities between gallbladder stones patients with and without cholecystectomy based on sex and the location of the CRC, we classified CRC into proximal colon cancer (ICD-9 code 153.0, 153.1, 153.4, and 153.6), distal colon cancer (ICD code 153.2, 153.3, and 153.7), and rectal cancer (ICD code 154.0 and 154.1). To address the concern of constant proportionality, we examined the proportional hazard model assumption using a test of scaled Schoenfeld residuals.

## 3. Results

The study included a cholecystectomy cohort of 83,963 GBS patients and a non-cholecystectomy cohort of 83,963 GBS patients, respectively (Table 1). The cholecystectomy and non-cholecystectomy cohorts were well matched for age, sex, occupation, urbanization level, and comorbidities. The mean ages in the cholecystectomy and non-cholecystectomy cohorts were 56.5 ± 17.1 and 56.8 ± 17.2 years, respectively. Most patients were aged ≥ 50 years (61.2%) and were men (54.2%). The top three common comorbidities according to the order of the frequency in these study cohorts were hypertension (21.7%), diabetes (13.6%), and cirrhosis (10.3%). It was noted that most cholecystectomy individuals were those engaged in a blue collar occupation or living in the lowest urbanization level.

**Figure 2:**
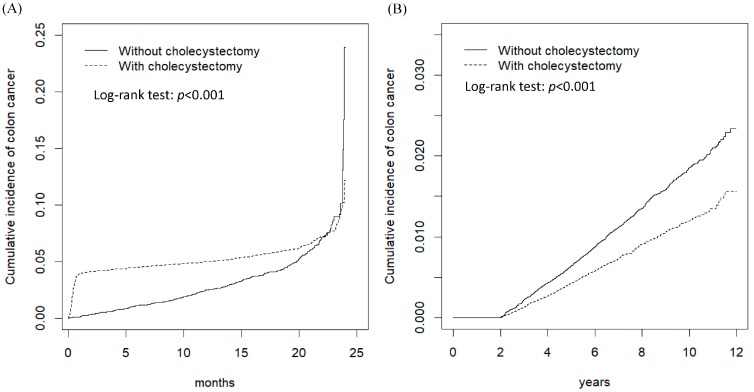
The cumulative incidence comparison of colon cancer for gallbladder stones patients with (dashed line) or without (solid line) cholecystectomy for the follow-up period ≤ 6 months (**A**) and for the follow-up period > 6 months (**B**).

Figure 2A shows the cumulative incidence of CRC within the first 6 months of the follow-up period was higher in the cholecystectomy cohort than that in the non-cholecystectomy cohort (log-rank test *p* < 0.001). However, Figure 2B shows the cumulative incidence of CRC after 6 months of the follow-up period was higher in the non-cholecystectomy cohort than that in the cholecystectomy cohort (log-rank test *p* < 0.001). The average follow-up duration was 5.53 ± 3.32 years for the cholecystectomy cohort and 6.81 ± 3.28 years for the non-cholecystectomy cohort, respectively.

Table 2 presents the comparison of the CRC incidence densities within the first 6 months of the follow-up period between GBS patients with and without cholecystectomy, based on the stratification of demographic characteristics and the presence or absence of a comorbidity. The overall incidence density rates of CRC in the cholecystectomy and non-cholecystectomy cohorts were 15.3 and 1.97 per 1000 person-years, respectively. Compared with patients without cholecystectomy, those with cholecystectomy were associated with an increased risk of CRC (adjusted HR (aHR) = 7.90, 95% CI = 6.27–9.94) after adjustment for age, hypertension, stroke, diabetes, CAD, and colorectal adenomas. The GBS patients with cholecystectomy had a higher risk of developing CRC during the initial 6 months of the follow-up in each subgroups of sex, age, occupation category, urbanization level, and the presence or absence of a comorbidity. In the model evaluating the colon cancer risk throughout the overall follow-up period, results of the test revealed a significant relationship between Schoenfeld residuals for cholecystectomy and the follow-up time <6 months, suggesting the proportionality assumption was violated. In the subsequent analyses, we stratified the follow-up duration to deal with the violation of proportional hazard assumption (Table 2). It was noted that the aHR of CRC for the cholecystectomy cohort was significantly higher in the first 3 months of follow-up (aHR = 15.5, 95% CI = 11.2–21.5), and then the risk reduced to be insignificant for 3–6 months of follow-up.

Table 3 presents the comparison of the CRC incidence densities after 6 months of the follow-up period between GBS patients with and without cholecystectomy, stratified by demographic characteristics and the presence or absence of a comorbidity. The overall incidence density rates of CRC in the cholecystectomy and non-cholecystectomy cohorts were 1.50 and 2.21 per 1000 person-years, respectively. Compared with patients without cholecystectomy, those with cholecystectomy were associated with a decreased risk of CRC (aHR = 0.66, 95% CI = 0.60–0.73) after adjustment for age, sex, hypertension, diabetes, chronic kidney injury, stroke, CAD, colorectal adenomas, and COPD. The GBS patients with cholecystectomy had a lower risk of developing CRC after 6 months of follow-up in each subgroups of sex, age, occupation category, urbanization level, and the presence or absence of a comorbidity. Results showed that there was no significant relationship between Schoenfeld residuals for cholecystectomy and the follow-up time >6 months (*p*-value = 0.16) in the model evaluating the colon cancer risk. It was noted that the aHR of CRC for the cholecystectomy cohort decreased with incremental duration of the follow-up, particularly for those who had more than 3 years of follow-up.

Table 4 presents the comparison of the colon cancer incidence densities between gallbladder stones patients with and without cholecystectomy, stratified by sex, location of cancer, and follow-up duration. Compared with the non-cholecystectomy cohort, the overall risks of CRC in each location were greater for those in the cholecystectomy cohort within the first 6 months of follow-up. Although the overall risks of CRC in each location were smaller for those in the cholecystectomy cohort after more than 6 months of follow-up, it was noted that the reduced risk of CRC for the cholecystectomy cohort was statistically significant only in rectal cancer after separately considering females and males.

## 4. Discussions

The prevalence of cholecystectomy was relatively higher in men (54.2%) and in patients older than 50 years (62.0%) with mean ages of 56.5 ± 17.1 years. In contrast to the predilection of women for cholecystectomy based on a hospitalization database from the United States, our findings were consistent with a hospitalization-based study conducted in Japan and Taiwan to demonstrate that most patients undergoing cholecystectomy were men [21,22]. Furthermore, the findings with the predisposition of patients aged ≥ 50 years to cholecystectomy were consistent with the liability of the elderly to GBS due to increased biliary cholesterol secretion, impaired bile salt synthesis, poor gallbladder contractility, and increased exposure to pro-lithogenic factors [23,24]. Whereas most individuals with cholecystectomy belonged to the occupation category of a blue collar occupation (52.6%) and lived in the least urbanized area (33.6%), which might be due to the relatively unhealthy lifestyle behaviors [25].

Hypertension, diabetes, and cirrhosis were the top three most common comorbidities in the cholecystectomy cohort. Hypertension impairs the gallbladder emptying due to enhancing the sympathetic tone [26]. Whereas diabetes increases biliary cholesterol secretion and impairs the gallbladder motility [27,28]. The association between cirrhosis and GBS may be bidirectional with increased secretion of bile pigments, impaired gallbladder emptying, and increased peripheral conversion of androgen to estrogen in cirrhotic patients; furthermore, increased hepatotropic virus infection and fatty liver disease are relatively common in patients with GBS [29]. It should be noted that there might be a surveillance bias for more comorbidities in the cholecystectomy cohort, which would skew the associations between some comorbidities with cholecystectomy, as there was no recommendation for implementing routine screening of comorbidities in asymptomatic GBS patients. Moreover, the literature did not support that GBS individuals with comorbidities would have more chance to develop symptoms and, therefore, necessitate cholecystectomy without the development of biliary complications or biliary colic [30,31].

It might raise a debate for the choice of presenting results before and after 6 months as it theoretically takes years for the development of a new-onset cancer [32]. Actually, we chose this point in time based on a consequence of patterns seen in the data. Moreover, the definition of synchronous cancer means two or more cancers identified at the same time or within 6 months. Our findings show that cholecystectomy was associated with an increased risk of CRC during the first 3 months of follow-up and then the risk decreased between 3 and 6 months of follow-up. Our study supports that the positive association between cholecystectomy and the CRC risk within the first 6 months of follow-up might be caused by a surveillance bias or the presence of pre-existing cancer. The cholecystectomy cohort, rather than the non-cholecystectomy cohort, might access more medical examinations to get diagnosed with incidental CRC during the initial periods of follow-up for cholecystectomy. Therefore, it is rational to exclude the detrimental effect of cholecystectomy on the development of CRC soon within 6 months.

Our results show that the risk of CRC in patients with GBS would decrease after cholecystectomy when the follow-up period was greater than 6 months. Moreover, the decreased risk of CRC in the cholecystectomy cohort after more than 6 months of follow-up was universal in each subgroup of sex, age, occupation category, urbanization level, and the presence or absence of a comorbidity. We note that the risk of CRC after cholecystectomy decreased with an incremental duration of follow-up, particularly for those with duration of follow-up greater than 3 years (Figure 2 and Table 3), and was particularly obvious in the groups of younger age and no comorbidity (Table 3). These findings support the cholecystectomy was associated with a decreased a risk of CRC in GBS patients although this observational study could not establish the causal relationship and protective mechanism. However, our findings show that the decreased risk is mainly on the development of rectal cancer (Table 4).

There are several pros and cons for the association of cholecystectomy with the development of CRC. The possible explanations for the positive association of cholecystectomy with CRC include the following: First, the exposure of carcinogenic bile acids to the bowels, particularly the proximal colon, will increase after cholecystectomy since the reabsorption of bile acids through enterohepatic circulation in the impaired [10,33]. Second, cholecystectomy can increase the risk of CRC by enhancing the risk of the metabolic syndrome due to the impairment of cholesterol metabolism [26,34,35]. Third, cholecystectomy can change the activity of colonic microbiota, which can also lead to the development of CRC [36,37]. However, our study does not demonstrate the detrimental effect of cholecystectomy on the development of CRC, therefore, these explanations above hardly support our findings.

On the other hand, the possible mechanisms for the inverse relationship between cholecystectomy and CRC in patients with GBS may be as noted in the following: First, lifestyle modification may be improved in the cholecystectomy cohort and the risk of CRC can be diminished consequently. Second, eliminating the effect of GBS on the development of CRC by removing an inflammatory gallbladder characterized by increased oxidative stress [9,10,38]. Third, decreasing the exposure time to GBS may decrease the risk of CRC, which is observed in our study. The mean exposure time of GBS was 2.16 ± 2.83 years, measured from the enrollment (diagnosis of GBS) to cholecystectomy, for the cholecystectomy cohort with CRC and was 4.46 ± 2.91 years, measured from the enrollment to the development of CRC, for the non-cholecystectomy cohort with CRC, respectively (data not shown). We acknowledge that the patient might have developed GBS before the enrollment and the exposure time could not be accurate. Therefore, the relationship between exposure time of GBS and the development of CRC cannot be ascertained based on our study. However, our study portends the importance of studying on the relationship of GBS exposure time to the risk of CRC. Moreover, the pathogenesis between right-side CRC and left-side CRC is different. Microsatellite instability is predominantly seen in right-sided CRC and chromosomal instability is predominantly seen in left-sided CRC, respectively [39,40]. It, therefore, requires more studies to clarify the mechanisms for the ameliorating effect of cholecystectomy on the development of rectal cancer, rather than the proximal or distal colon, which was observed in our study.

Our study has several strengths. First, our study is the largest population-based cohort study to assess the risk of CRC after cholecystectomy for GBS patients [41,42]. Additionally, this longitudinal database with a 12-year observation period provides the temporal association between cholecystectomy and the subsequent development of CRC. Second, the Taiwan NHI program is a mandatory insurance system and it almost has provided health care for every resident in Taiwan, therefore, the findings of this study provide the generalizability in Taiwan. Since NHIRD is a government-managed database, the proportion of missing data is low. We only analyzed the complete data as shown in Figure 1. Third, the possible bias for misclassifying asymptomatic GBS into general population will be mitigated by the comparison of the CRC risk between the GBS patients with and without cholecystectomy, rather than between the GBS patients and the general population. Fourth, assessing the effect of post-cholecystectomy duration on the risk of CRC can also diminish the bias related to temporal effect. However, our study cannot assure that more exposure time of GBS would increase the risk for the development of CRC since the patient might have GBS, either symptomatic or asymptomatic, before the diagnosis was registered in the NHIRD. Fifth, we analyzed the interaction between cholecystectomy, sex, and location of CRC to cross-check the effect of cholecystectomy on the development of CRC. The study conducted in Europe, where CRC incidence is higher in men with GBS, shows that GBS is closely related to the development of CRC in female, but not in men [43]. However, our findings consistently demonstrated that cholecystectomy was associated with a decreased risk of rectal cancer in both genders. We performed a lag analysis to avoid the reverse causality effect and found the risk of CRC for the cholecystectomy cohort was only significantly higher in the first 3 months of follow-up. Moreover, the rerun Cox regression models (stratifying the follow-up periods into 6–12 months, 1–3 years, 3–6 years, and >6 years) support the inverse relationship between cholecystectomy and CRC, particularly for those who had more than 3 years of follow-up. The covariates considered for multivariable analysis include age, gender, hypertension, diabetes mellitus, chronic kidney diseases, stroke, CAD, colorectal adenomas, and COPD. However, we did not separately perform a modeling test for the covariates above because the evidence supporting their predisposing to CRC through the development of GBS remained weak and inconclusive.

Our study also has several limitations. First, the dietary habits, alcohol drinking, smoking, physical activity, and history of total parenteral nutrition linking between GBS and CRC were unavailable from the insurance claims database even though we used the diagnosis of obesity instead of body mass index, COPD instead of smoking, and alcohol-related illness instead of alcohol drinking. Second, we could not individually validate each diagnosis claim although the Taiwan government has audited all insurance claims for medical reimbursement. However, the data regarding GBS, cholecystectomy, and CRC should be more reliable because their diagnostic criteria were specific. In Taiwan, the diagnosis of gallstones should be made according to the image-evidenced stones within the gallbladder and the government only allows the reimbursement for cholecystectomy after auditing the indications for biliary complications and the acquisition of surgical specimens. Based on the Taiwan Cancer Registry Database and medical records, the government waives copayment for a patient with Catastrophic Illness Certification of malignancy. Furthermore, a substantial concordance between the claims data and the patients’ interview in NHIRD has been described in the literature [44,45]. Finally, this observational study could not ascertain the causal relationship and protective mechanism of cholecystectomy for the development of CRC.

More than 80% of GBS will remain symptom-free and only 3% of the symptomatic GBS will develop acute cholecystitis; therefore, prophylactic cholecystectomy is not indicated for asymptomatic GBS [21,22,46]. The ameliorating effect of cholecystectomy on the development of CRC may be underappreciated in the former studies due to erroneous comparison with the general population [12,13,14]. However, we could not recommend prophylactic cholecystectomy for the prevention of CRC simply based on our observational study since the number needed to treat (NNT) per year (6 months or longer after cholecystectomy) for cholecystectomy for preventing one CRC was 1408 (1000/2.21–1.50) in our study. Before recommending prophylactic cholecystectomy against the development of CRC, more studies are required to clarify the casual relationship, protective mechanism, and the cost–benefit effect.

## 5. Conclusions

Cholecystectomy is associated with a decreased risk of rectal cancer, rather than proximal or distal colon cancer, when the follow-up period was greater than 6 months. The positive association between cholecystectomy and the CRC risk within the first 6 months after cholecystectomy might be due to detection bias or the presence of pre-existing CRC. We could not ascertain the protective pathophysiology of cholecystectomy against CRC, either for the GBS or the general population, in this observational study.

## Figures and Tables

**Table 1:** Characteristics of patients between gallbladder stones patients with cholecystectomy and without cholecystectomy.

Variables	Cholecystectomy				*p*-Value
	Yes		No		
	(*N* = 83,963)		(*N* = 83,963)		
	*n*	%	*n*	%	
Age, Year					0.003
≤49	32,560	38.8	31,962	38.1	
50–64	21,334	25.4	21,316	25.4	
≥ 65	30,069	35.8	30,685	36.6	
Mean (SD) ^#^	56.5	17.1	56.8	17.2	0.002
Gender					0.42
Female	38,434	46.0	38,599	46.0	
Male	45,529	54.2	45,364	54.0	
Occupation					0.12
White collar	19,844	23.6	19,825	23.6	
Blue collar	44,186	52.6	43,854	52.2	
Others ^‡^	19,933	23.7	20,284	24.2	
Urbanization Level ^†^					0.63
1 (highest)	18,136	21.6	18,002	21.4	
2	24,031	28.6	24,103	28.7	
3	13,628	16.2	13,506	16.1	
4(lowest)	28,168	33.6	28,352	33.8	
Comorbidity					
Hypertension	18,197	21.7	18,663	22.2	0.01
Hyperlipidemia	3713	4.42	3645	4.34	0.42
Cirrhosis	8639	10.3	8497	10.1	0.25
Stroke	5636	6.71	5730	6.82	0.36
CAD	7405	8.82	7577	9.02	0.14
Diabetes mellitus	11,400	13.6	11,452	13.6	0.71
COPD	4061	4.84	4904	5.84	0.001
Chronic kidney diseases	3217	3.83	3216	3.83	0.99
Alcohol-related illness	1464	1.74	1357	1.62	0.04
Colorectal adenomas	280	0.33	290	0.35	0.67
Inflammatory bowel disease	112	0.13	188	0.22	0.001
Irritable bowel syndrome	89	0.11	100	0.12	0.42
Obesity	26	0.03	28	0.03	0.79

Chi-square test; ^#^: *t*-test; ^†^: The urbanization level was categorized by the population density of the residential area into 4 levels, with level 1 as the most urbanized and level 4 as the least urbanized. ^‡^: Other occupations included primarily retired, unemployed, or low-income populations.

**Table 2:** The comparison of the colon cancer incidence densities within the first 6 months of the follow-up period between gallbladder stones patients with and without cholecystectomy, based on the stratification of demographic characteristics and the presence or absence of a comorbidity.

Variables	Cholecystectomy						Crude HR (95% CI)	Adjusted HR ^§^ (95% CI)
	Yes			No				
	Event	PY	Rate ^#^	Event	PY	Rate ^#^		
All	623	40,812	15.3	82	41,702	1.97	7.68 (6.10, 9.67) ***	7.90 (6.27, 9.94) ***
Gender								
Female	275	18,778	14.6	30	19,203	1.56	9.29 (6.37, 13.5) ***	9.75 (6.69, 14.2) ***
Male	348	22,034	15.8	52	22,499	2.31	6.75 (5.04, 9.03) ***	6.86 (5.13, 9.18) ***
Age, Year								
≤49	34	16,112	2.11	4	15,953	0.25	8.39 (2.98, 23.7) ***	8.23 (2.92, 23.2) ***
50–64	160	10,476	15.3	15	10,614	1.41	10.7 (6.32, 18.2) ***	10.7 (6.32, 18.2) ***
≥65	429	14,224	30.2	63	15,135	4.16	7.08 (5.44, 9.23) ***	7.10 (5.45, 9.25) ***
Occupation								
White collar	75	9795	7.66	7	9887	0.71	10.8 (4.96, 23.4) ***	10.4 (4.80, 22.6) ***
Blue collar	392	21,429	18.3	47	21,775	2.16	8.36 (6.18, 11.3) ***	8.63 (6.38, 11.7) ***
Others ^‡^	156	9588	16.3	28	10,039	2.79	5.76 (3.85, 8.61) ***	5.94 (3.97, 8.88) ***
Urbanization Level ^†^								
1 (highest)	115	8905	12.9	16	8957	1.79	7.19 (4.26, 12.1) ***	7.27 (4.31, 12.3) ***
2	156	11,707	13.3	20	11,982	1.67	7.91 (4.97, 12.6) ***	8.05 (5.06, 12.8) ***
3	96	6625	14.5	13	6705	1.94	7.39 (4.14, 13.2) ***	7.71 (4.32, 13.8) ***
4(lowest)	256	13,574	18.9	33	14,058	2.35	7.91 (5.51, 11.4) ***	8.16 (5.68, 11.7) ***
Comorbidity ^&^								
No	283	25,098	11.3	41	25,777	1.61	6.95 (5.01, 9.64) ***	7.12 (5.13, 9.89) ***
Yes	340	15,714	21.6	41	15,925	2.52	8.44 (6.10, 11.7) ***	8.62 (6.24, 11.9) ***
Follow-Up Period								
<3 months	589	20,574	28.6	39	20,923	1.86	15.2 (11.0, 21.1) ***	15.5 (11.2, 21.5) ***
3–6 months	34	40,375	0.84	43	41,484	1.04	0.81 (0.52, 1.27)	0.85 (0.54, 1.33)

PY, person-years; Rate ^#^, incidence rate per 1000 person-years. Crude hazard ratio (HR), relative hazard ratio. Adjusted HR ^§^ multivariable analysis including age, comorbidities of hypertension, stroke, diabetes mellitus, CAD, and colorectal adenomas. ^†^: The urbanization level was categorized by the population density of the residential area into 4 levels, with level 1 as the most urbanized and level 4 as the least urbanized. ^‡^ Other occupations included primarily retired, unemployed, or low-income populations. ^&^ Individuals with any comorbidity of hypertension, hyperlipidemia, cirrhosis, stroke, coronary artery disease, diabetes mellitus, chronic obstructive pulmonary disease (COPD), chronic kidney diseases, alcohol-related illness, colorectal adenomas, inflammatory bowel disease, irritable bowel syndrome, and obesity were classified into the comorbidity group; *** *p* < 0.001.

**Table 3:** The comparison of the colon cancer incidence densities after 6 months of the follow-up period between gallbladder stones patients with and without cholecystectomy, stratified by demographic characteristics and the presence or absence of a comorbidity.

Variables	Cholecystectomy						Crude HR (95% CI)	Adjusted HR ^§^ (95% CI)
	Yes			No				
	Event	PY	Rate ^#^	Event	PY	Rate ^#^		
All	638	423,992	1.50	1170	530,121	2.21	0.69 (0.63, 0.76) ***	0.66 (0.60, 0.73) ***
Gender								
Female	253	200,207	1.26	487	262,402	1.86	0.69 (0.59, 0.80) ***	0.66 (0.57, 0.77) ***
Male	385	223,786	1.72	683	267,719	2.55	0.68 (0.60, 0.77) ***	0.66 (0.58, 0.75) ***
Age, Year								
≤49	53	176,539	0.30	231	235,703	0.98	0.33 (0.25, 0.45) ***	0.33 (0.24, 0.45) ***
50–64	177	113,021	1.57	355	139,776	2.54	0.63 (0.52, 0.75) ***	0.62 (0.52, 0.75) ***
≥65	408	134,432	3.03	584	154,642	3.78	0.81 (0.71, 0.91) ***	0.80 (0.71, 0.91) ***
Occupation								
White collar	227	161,496	1.41	227	161,496	1.41	0.53 (0.41, 0.69) ***	0.53 (0.41, 0.69) ***
Blue collar	651	267,670	2.43	651	267,670	2.43	0.71 (0.63, 0.81) ***	0.70 (0.62, 0.79) ***
Others ^‡^	292	100,955	2.89	292	100,955	2.89	0.65 (0.54, 0.79) ***	0.66 (0.54, 0.79) ***
Urbanization Level ^†^								
1 (highest)	108	94,205	1.15	251	126,211	1.99	0.59 (0.47, 0.74) ***	0.57 (0.45, 0.71) ***
2	165	123,195	1.34	343	157,654	2.18	0.62 (0.52, 0.75) ***	0.59 (0.49, 0.71) ***
3	109	68,301	1.60	205	85,985	2.38	0.69 (0.55, 0.87) **	0.66 (0.53, 0.84) ***
4(lowest)	256	138,291	1.85	371	160,270	2.31	0.80 (0.68, 0.94) **	0.79 (0.67, 0.92) **
Comorbidity ^&^								
No	313	278,547	1.12	715	362,620	1.97	0.58 (0.51, 0.66) ***	0.55 (0.48, 0.63) ***
Yes	325	145,445	2.23	455	167,501	2.72	0.83 (0.72, 0.95) **	0.82 (0.71, 0.95) **
Follow-Up Period								
6–12 months	56	39,942	1.40	84	41,114	2.04	0.69 (0.49, 0.96) *	0.70 (0.50, 0.99) *
1–3 years	203	138,722	1.46	303	151,779	2.00	0.73 (0.61, 0.88) ***	0.74 (0.62, 0.88) ***
3–6 years	211	142,551	1.48	393	180,127	2.18	0.68 (0.58, 0.80) ***	0.64 (0.54, 0.76) ***
>6 years	168	102,778	1.63	390	157,099	2.48	0.66 (0.55, 0.79) ***	0.60 (0.50, 0.73) ***

PY, person-years; Rate ^#^, incidence rate per 1000 person-years. Crude HR, relative hazard ratio. Adjusted HR. ^§^ multivariable analysis including age, gender, comorbidities of hypertension, diabetes mellitus, chronic kidney diseases, stroke, CAD, colorectal adenomas, and COPD. ^†^: The urbanization level was categorized by the population density of the residential area into 4 levels, with level 1 as the most urbanized and level 4 as the least urbanized. ^‡^ Other occupations included primarily retired, unemployed, or low-income populations. ^&^ Individuals with any comorbidity of hypertension, hyperlipidemia, cirrhosis, stroke, coronary artery disease, diabetes mellitus, COPD, chronic kidney diseases, alcohol-related illness, colorectal adenomas, inflammatory bowel disease, irritable bowel syndrome, and obesity were classified into the comorbidity group; * *p* < 0.05, ** *p* < 0.01, *** *p* < 0.001.

**Table 4:** The comparison of the colon cancer incidence densities between gallbladder stones patients with and without cholecystectomy, stratified by sex, location of cancer, and follow-up duration.

Outcome	Cholecystectomy				Crude HR (95% CI)	Adjusted HR ^§^ (95% CI)
	Yes		No			
	Event	Rate ^#^	Event	Rate ^#^		
Follow-Up Period ≤ 6 Months						
All						
Proximal colon cancer	90	2.21	9	0.22	10.1 (5.09, 20.0) ***	10.4 (5.23, 25.6) ***
Distal colon cancer	52	1.27	7	0.17	7.51 (3.41, 16.5) ***	7.70 (3.50, 17.0) ***
Rectal cancer	102	2.50	13	0.31	7.93 (4.45, 14.1) ***	8.06 (4.53, 14.4) ***
Female						
Proximal colon cancer	49	2.61	4	0.21	12.4 (4.48, 34.4) ***	13.0 (4.69, 36.0) ***
Distal colon cancer	22	1.17	3	0.16	7.43 (2.23, 24.8) ***	7.73 (2.31, 25.8) ***
Rectal cancer	43	2.29	2	0.10	21.8 (5.28, 89.9) ***	22.5 (5.46, 93.0) ***
Male						
Proximal colon cancer	44	2.00	9	0.40	4.94 (2.41, 10.1) ***	4.99 (2.44, 10.2) ***
Distal colon cancer	37	1.68	27	1.20	1.40 (0.85, 2.29)	1.42 (0.86, 2.33)
Rectal cancer	81	3.68	42	1.87	1.96 (1.35, 2.85) ***	2.00 (1.38, 2.91) ***
Follow-Up Period > 6 Months						
All						
Proximal colon cancer	70	0.17	121	0.23	0.77 (0.57, 1.03)	0.72 (0.54, 0.97) *
Distal colon cancer	93	0.22	173	0.33	0.70 (0.55, 0.90) **	0.65 (0.51, 0.84) ***
Rectal cancer	149	0.35	289	0.55	0.66 (0.54, 0.81) ***	0.63 (0.51, 0.77) ***
Female						
Proximal colon cancer	35	0.17	59	0.22	0.82 (0.54, 1.24)	0.76 (0.50, 1.15)
Distal colon cancer	32	0.16	62	0.24	0.70 (0.46, 1.08)	0.66 (0.43, 1.02)
Rectal cancer	56	0.28	116	0.44	0.67 (0.48, 0.92) *	0.64 (0.46, 0.88) **
Male						
Proximal colon cancer	32	0.14	58	0.22	0.71 (0.46, 1.10)	0.68 (0.44, 1.04)
Distal colon cancer	54	0.24	88	0.33	0.79 (0.56, 1.11)	0.73 (0.52, 1.03)
Rectal cancer	71	0.32	142	0.53	0.63 (0.47, 0.84) **	0.59 (0.44, 0.79) ***

PY, person-years; Rate ^#^, incidence rate per 1000 person-years. Crude HR, relative hazard ratio. Only those found to be significant in the univariable analysis were further examined in the multivariable analysis. Adjusted HR ^§^ multivariable analysis including age, gender, and comorbidities. * *p* < 0.05, ** *p* < 0.01, *** *p* < 0.001.

## Abbreviations

CRC	colorectal cancer
GBS	gallbladder stones
NHIRD	National Health Insurance Research Database
aHR	adjusted hazard ratio
CI	confidence interval
NHI	National Health Insurance
NHRI	National Health Research Institute
IRB	Institutional Review Board
CAD	coronary artery disease
COPD	chronic obstructive pulmonary disease
ICD-9-CM	International Classification of Diseases, Ninth Edition, Clinical Modification
SDs	standard deviations
HRs	hazard ratios
NNT	number needed to treat
